# Sphingosine-1-Phosphate Receptor 5 Modulates Early-Stage Processes during Fibrogenesis in a Mouse Model of Systemic Sclerosis: A Pilot Study

**DOI:** 10.3389/fimmu.2017.01242

**Published:** 2017-09-29

**Authors:** Katrin G. Schmidt, Martina Herrero San Juan, Sandra Trautmann, Lucija Berninger, Anja Schwiebs, Florian M. Ottenlinger, Dominique Thomas, Frank Zaucke, Josef M. Pfeilschifter, Heinfried H. Radeke

**Affiliations:** ^1^pharmazentrum frankfurt/ZAFES, Institute of Pharmacology and Toxicology, Hospital of the Goethe University, Frankfurt, Germany; ^2^pharmazentrum frankfurt/ZAFES, Institute for Clinical Pharmacology, Hospital of the Goethe University, Frankfurt, Germany; ^3^Dr Rolf M Schwiete Research Unit for Osteoarthritis, Orthopedic University Hospital, Friedrichsheim gGmbH, Frankfurt, Germany

**Keywords:** systemic sclerosis, sphingosine-1-phosphate receptor 5, sphingolipid sphingosine-1-phosphate, sphingolipids, inflammation, bleomycin, fibrogenesis, mouse model

## Abstract

Systemic sclerosis (SSc) is a rare multi-organ autoimmune disease characterized by progressive skin fibrosis. Inflammation, type 2 immunity, and fibrogenic processes are involved in disease development and may be affected by sphingolipids. However, details about early-stage pathophysiological mechanisms and implicated mediators remain elusive. The sphingolipid sphingosine-1-phosphate (S1P) is elevated in the sera of SSc patients, and its receptor S1P5 is expressed in skin tissue. Nevertheless, almost nothing is known about the dermatological contribution of S1P5 to inflammatory and pro-fibrotic processes leading to the pathological changes seen in SSc. In this study, we observed a novel effect of S1P5 on the inflammatory processes during low-dose bleomycin (BLM)-induced fibrogenesis in murine skin. By comparing 2-week-treated skin areas of wild-type (WT) and S1P5-deficient mice, we found that S1P5 is important for the transcriptional upregulation of the Th2 characteristic transcription factor *GATA-3* under treatment-induced inflammatory conditions, while *T-bet* (Th1) and *FoxP3* (Treg) mRNA expression was regulated independently of S1P5. Additionally, treatment caused a regulation of *S1P receptor 1* and *S1P receptor 3* mRNA as well as a regulation of long-chain ceramide profiles, which both differ significantly between the genotypes. Despite S1P5-dependent differences regarding inflammatory processes, similar macroscopic evidence of fibrosis was detected in the skin histology of WT and S1P5-deficient mice after 4 weeks of subcutaneous BLM treatment. However, at the earlier 2-week point in time, the mRNA data of *pro-collagen type 1* and *SMAD7* indicate a pro-fibrotic S1P5 contribution in the applied SSc mouse model. In conclusion, we propose that S1P5 plays a role as a novel modulator during the early phase of BLM-caused fibrogenesis in murine skin. An immediate relationship between dermal S1P5 expression and fibrotic processes leading to skin alterations, such as formative for SSc pathogenesis, is indicated but should be studied more profound in further investigations. Therefore, this study is an initial step in understanding the role of S1P5-mediated effects during early stages of fibrogenesis, which may encourage the ongoing search for new therapeutic options for SSc patients.

## Introduction

Systemic sclerosis (SSc, also known as scleroderma) is a rare multi-organ autoimmune disease with a complex pathophysiology, characterized by microvascular damage, deregulated immunity, and fibrosis. However, etiology and pathogenesis of SSc are still elusive (1, 2).

Skin fibrosis is one important hallmark of SSc and especially the extent of skin involvement and its rate of progression reveal the severity of internal organ complications (3, 4). Once established, an irreversible and progressive fibrotic organ failure is responsible for a high mortality. Fibrotic skin thickening, expansion of connective tissue, and resulting tissue dysfunctions are the consequence of remodeling processes with an excessive deposition of extracellular matrix components such as type I collagen (2, 5).

In recent years, several factors have been proposed as markers indicating pathophysiological events in SSc patients. For instance, CXCL4, CD146, dickkopf-related protein 1, lysyl oxidase, and cartilage oligomeric matrix protein (COMP) are discussed (6–10). Overexpressed COMP assists skin-fibrotic SSc features in two ways: intracellular COMP auxiliary collagen secretion, while extracellular COMP acts as crosslinker between collagen I and collagen XII and therefore its presence results in a more compact extracellular matrix network (10–13).

Moreover, the bioactive sphingolipid sphingosine-1-phosphate (S1P) may be causatively related to abnormalities described in vasculature, immune system, and connective tissues of SSc patients (14). In support, variations of circulating S1P levels have been detected in various fibrotic diseases including SSc (15–17). S1P may modulate several processes contributing to fibrosis such as angiogenesis, alternation of lymphocyte trafficking, and trans-activation of the transforming growth factor beta (TGF-β)/SMAD pathway (18–21). With respect to processes induced by extracellular S1P, the type of targeted S1P receptor is decisive. One particularly interesting receptor of the five existing S1P receptors, named as S1P1 to S1P5, is S1P receptor 5 (S1P5). S1P5 affects proliferation, migration, has recently shown to be involved during early TGF-β-induced processes, and is expressed in the skin (22–25). However, current knowledge regarding S1P5 function is limited and almost nothing is known about its involvement during fibrogenesis.

Fibrotic extracellular matrix alterations originate from earliest events of inflammation and vascular injury, which includes a reduction of capillaries (26, 27). In early-stage inflammatory processes, damage-induced upregulation of intercellular adhesion molecule 1 (ICAM-1) on activated endothelial cells mediates tissue infiltration of different immune cells (28). These together create a fibrogenic inflammatory signature. Among others, T cells and macrophages represent a main part of the cellular infiltrate. Especially an exaggerated Th2 response as well as the presence of alternatively activated M2 macrophages and mast cells trigger the production of fibrogenic cytokines such as TGF-β. TGF-β is known as a key pro-fibrotic cytokine mediating the production of collagens, COMP, and the differentiation of alpha-smooth muscle actin (α-SMA) expressing myofibroblasts *via* SMAD or non-canonical signaling cascades (29–31). Subsequently, TGF-β signaling provokes typical pro-fibrotic modifications in SSc (13). Detection of autoantibodies directed against endothelial antigens and others, as well as T cell alterations in sera of SSc patients, supports the assumption that an impaired regulation of the immune system drives SSc pathogenesis (32–34).

A possible trigger for disease onset is a primary tissue injury caused, for example, by autoimmunity or extrinsic agents, which activate the immune system and initiate an inflammatory response. For the protection of the skin against penetrating extrinsic agents, the epidermal permeability barrier is of great importance. Accordingly, a functional disturbance obtained through variations in the extracellular lipid composition of the outer epidermal cell layer may result in disease. In this context, primarily the sphingolipid-species ceramide (Cer) and glucosylceramide (GluCer) play an important role in barrier function (35–38). Furthermore, accumulation of GluCer in macrophages is associated with inflammatory diseases (39).

However, data concerning fibrotic S1P5 involvement are rare and ambiguous. In this pilot study, we investigated the contribution of S1P5-mediated effects to early-stage processes driving cutaneous fibrosis, in a modified mouse model of scleroderma. Our results clearly demonstrate that low-dose bleomycin (BLM) induces S1P5-dependent variations in cutaneous Cer and GluCer profiles, as well as a transcriptional upregulation of inflammation-associated factors like Th2 transcription factor *GATA-3* and *S1P3*, as well as pro-fibrotic collagen type I alpha 1 (*COL1A1*) specifically in the early sclerotic phase. Thus, our data point to a modulating involvement of S1P5 during early-stage cutaneous processes, potentially promoting the pathogenesis of SSc.

## Materials and Methods

### Mice and Reagents

Bleomycin sulfate was purchased from Medac (Wedel, Germany) and dissolved in phosphate-buffered saline (PBS) to a final concentration of 250 µg/ml. After sterile filtration, aliquots in daily needed quantities were stored at −20°C until further application. Safe handling and disposal of hazardous BLM were performed according to the Safety Data Sheet.

C57BL/6J mice were obtained from Janvier (Le Genest-Saint-Isle, France). The S1P5-deficient mouse strain was generated (40) and kindly provided by The Scripps Research Institute (La Jolla, CA, USA).

The animals were bred and fed under pathogen-free conditions in secluded scantainers. All animal experiments were performed in accordance with the German animal welfare law and had been declared to the Animal Welfare Officer as the chairperson of the ethical oversight committee of the Goethe University Frankfurt/Main. The animal housing facility was licensed by the local authorities of the Regierungspraesidium Darmstadt (Az: 32.62.1). The methods used to euthanize the animals humanely were consistent with the recommendations of the AVMA Guidelines for the Euthanasia of Animals.

### Experimental Animal Model of BLM-Induced Scleroderma

In the experiment, 100 µl of BLM (250 µg/ml) or PBS were injected subcutaneously into the shaved upper back areas of 10- to 14-week-old female wild-type (WT) C57BL/6J and S1P5-deficient (S1P5^−/−^) mice once daily for 2 or 4 weeks (5 days/week). The injection area was freshly shaved weekly.

### Sample Collection

Mice were anesthetized, sacrificed, and the remaining fur was carefully shaved off at the site of injection. These skin areas were removed, cut into pieces, and stored under the conditions necessary for the respective following methods of analysis. For histology, skin tissues were fixed with 4% (*v/v*) formaldehyde solution (Roti^®^-Histofix, Carl Roth, Karlsruhe) overnight and were subsequently transferred into 1% formaldehyde solution diluted in PBS for 6 h. Tissue pieces for RNA were secured in RNA later (Qiagen, Hilden), incubated overnight at 4°C and stored at −80°C. Samples for lipid analysis were immediately frozen in liquid nitrogen and stored at −80°C.

### Histopathology

Formaldehyde-fixed skin tissues were dehydrated by rising concentrations of ethanol and xylol (automatic tissue processor, Leica, Wetzlar). After embedding in paraffin wax, skin tissues were cut into 4-µm sections (embedding station EG 1150, RM 2235 microtome, Leica, Wetzlar), placed onto microscope slides (Menzel, Braunschweig), and dried at room temperature overnight.

*Hematoxylin and eosin (H&E) staining*. Deparaffinized sections were stained with Mayer’s hematoxylin solution (Applichem, Darmstadt), counterstained with 0.5% Eosin G solution (Carl Roth, Karlsruhe) and covered with Aquatex (Merck, Darmstadt).

*Masson’s trichrome staining*. Deparaffinized sections were stained with acetic 0.1% Azocarmine G solution (Fluka, Seelze) and differentiated in 0.1% acetic acid. Following incubation in 5% phosphowolframic acid (Fluka, Seelze) and counterstaining with an acid dye (1:1) mixture consisting of aniline blue (Sigma-Aldrich, Darmstadt) and Orange G (Sigma-Aldrich, Darmstadt), the sections were differentiated in 96% ethanol, dehydrated by ethanol and xylol, and finally covered with Entellan (Merck, Darmstadt).

*Immunohistochemical staining for CD31 and α-SMA*. After deparaffinization, antigen retrieval (Dako, Jena) and blocking (protein block, Dako, Jena), skin sections were incubated overnight at 4°C with the primary antibody (rabbit anti-CD31, ab28364, abcam, Cambridge; α-smooth muscle – alkaline phosphatase antibody; A5691, Sigma, St. Louis, CA, USA). Detection of CD31 was performed with a specific anti-rabbit secondary antibody (N-Histofine, Nichirei Bioscience, Tokyo) and peroxidase conjugate (Vector DAB, Vector Laboratories, Burlingame, CA, USA). α-SMA detection was conducted by Permanent Red (Permanent AP Red Kit, Zytomed Systems, Berlin). Following counterstaining with Mayer’s hematoxylin solution, skin sections were covered with Aquatex.

*Immunohistochemical staining for COMP*. Deparaffinized and hyaluronidase-treated (Sigma-Aldrich, Steinheim) sections were blocked with 1% BSA/goat serum and subsequently incubated overnight at 4°C with a primary antibody rabbit anti-COMP (Immundiagnostik, Bensheim). A biotinylated anti-rabbit secondary antibody and red alkaline phosphatase conjugate were used (Vectastain ABC Kit, Vector Laboratories, Burlingame, CA, USA) for detection. Skin sections were subsequently counterstained with Mayer’s hematoxylin solution.

### Histopathological Evaluation

Dermal thickness was determined microscopically by evaluation of the skin sections stained beforehand with H&E and Masson’s trichome (BZanalyser software, Keyence, Osaka, Japan). The distance between epidermis–dermis and dermis–subcutis borders was determined by measurement at 15 points per/staining/and mouse using ImageJ software (open source) (Figure 2B).

Sample quality and the proportion of connective tissue in the skin were evaluated in a blinded fashion by three researchers based on overall images of Masson’s trichrome-stained skin sections. The dermis-to-subcutis ratios, as well as the extent of blue-stained collagenous connective tissue in the subcutis, were numerically scored with a previously described scoring system (Table 1). The total of these two scored parameters was used for evaluation of the expansion of connective tissue in the skin (Figure 2C).

**Table 1:** Scoring system to evaluate the expansion of collagenous connective tissue in the skin.

Score	Ratio of dermis to subcutis	Extent of connective tissue in the subcutis
1	1:2<	Non-existent
2	1:2	Little
3	1:1	Moderate
4	2:1	Intense
5	>2:1	–

*The ratio of dermis to subcutis as well as the extent of blue-stained collagenous connective tissue in the subcutis were numerically scored by three independent persons according to the above-mentioned and previously described scoring system. The total of both parameters was named as expansion of connective tissue, numerically expressed in a scoring range between 2 and 9 (Figure 2C)*.

**Figure 1:**
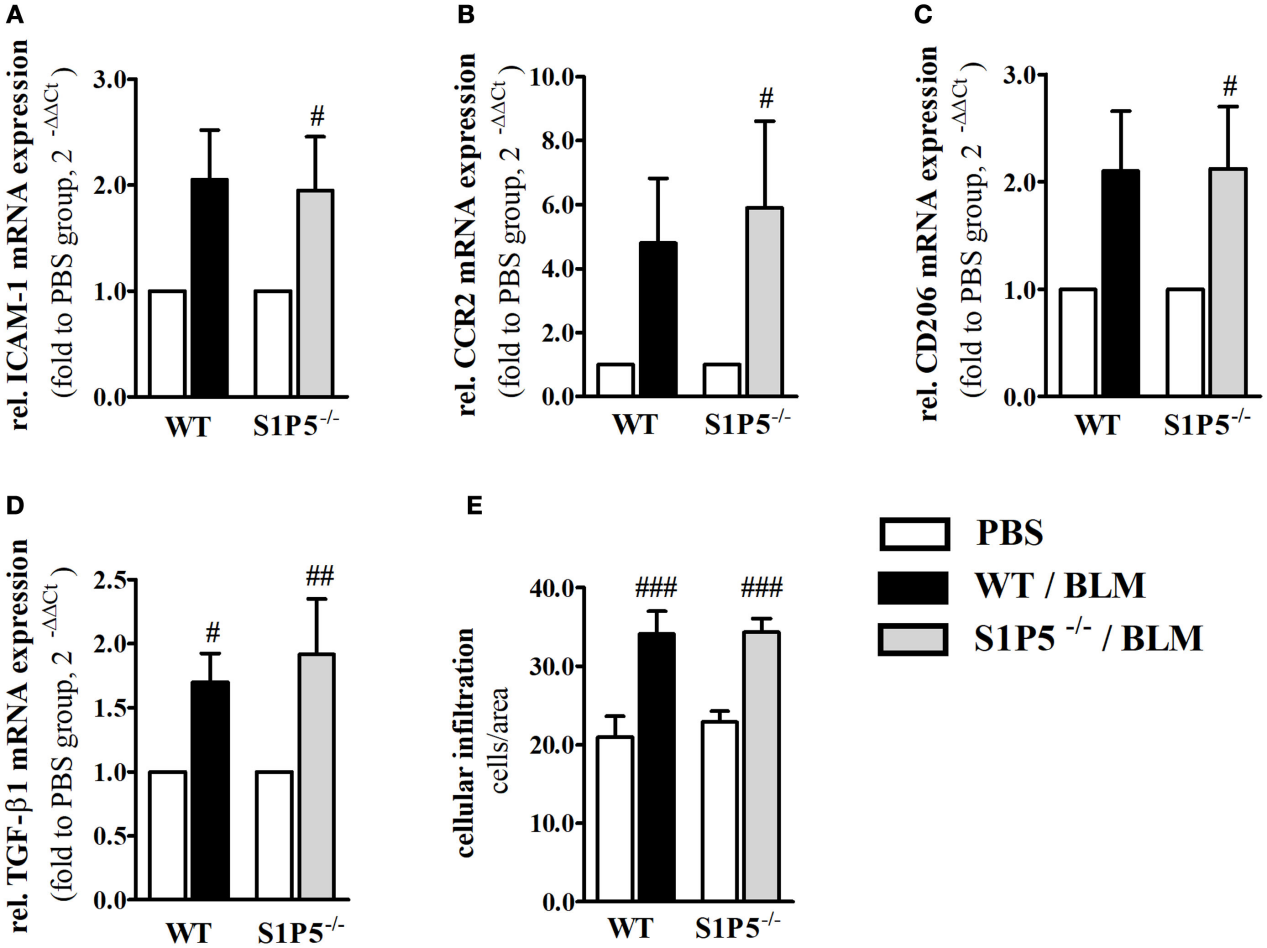
Markers of skin inflammation increased after 2 weeks of low-dose BLM treatment. Relative mRNA expression of **(A)** *ICAM-1*, **(B)** *CCR2*, **(C)** M2-makrophage marker *CD206*, and **(D)** *TGF-β1* in skin tissue of BLM-treated WT (black bars) and S1P5^−/−^ (gray bars) mice. The number of tissue-infiltrating cells detected in H&E-stained skin sections is shown in part **(E)**. mRNA expression levels were determined by qRT-PCR analysis and mRNA data are presented as fold change compared with the mean of the respective PBS controls. All data are shown as mean ± SD of *n* = 3–5 mice/group with # indicating *p* ≤ 0.05, ## for *p* ≤ 0.01 and ### for *p* ≤ 0.001, compared with the respective PBS controls (adjacent white bars). Statistical analysis was performed using a *one-sample t-test* **(A–D)** or a *one-way ANOVA with Bonferroni’s multiple comparison test* **(E)**. (rel. = relative). ANOVA, analysis of variance; BLM, bleomycin; H&E, hematoxylin and eosin; ICAM-1, intercellular adhesion molecule 1; PBS, phosphate-buffered saline; qRT-PCR, quantitative real-time polymerase chain reaction; WT, wild type.

Tissue-infiltrating cells, CD31-positive blood vessels, and α-SMA-positive myofibroblasts were counted in several areas similar in size per H&E-stained/CD31-marked/or α-SMA-marked skin sections by microscopical observations (Figures 1E and 4B; Figure S1 in Supplementary Material).

Cartilage oligomeric matrix protein expression within the dermis has been analyzed excluding regions of hair follicles (41) (ImageJ software, open source) (Figure 8A). The proportion of COMP was quantified as percentage of COMP-stained areas in relation to the total field of analysis. For consistency, all images were acquired (Axioscope2 Microscope, Zeiss, Jena) and analyzed on 1 day in each case.

### Isolation and Analysis of RNA by Real-time Polymerase Chain Reaction (RT-PCR)

Frozen skin biopsies were transferred to 1 ml TRIzol reagent (Invitrogen, CA, USA) and fully disrupted using a Tissue Ruptor (Qiagen, Hilden). Isolation of RNA was performed according to the manufacturer’s recommendations and RNA concentration was measured in duplicates using the Nano-Drop (Thermo Scientific, Dreieich). Equal RNA amounts were transcribed into cDNA by reverse transcriptase with a high-capacity cDNA reverse transcription kit including an RNase inhibitor (Life Technologies, CA, USA). The reverse transcription was executed with a RT-PCR program (25°C, 10 min, 37°C, 120 min, 85°C, 5 min).

TaqMan^®^ gene expression assays were performed in duplicates for every sample with Precision FAST 2× qPCR Master Mix (BioRad, Hercules, CA, USA). The quantitative RT-PCR was run at 95°C for 2 min and 50 times at 95°C for 5 s, 60°C for 20 s with the 7500 Fast Real-Time PCR System (Applied Biosystems, CA, USA). Similarly, 5′- FAM-tagged, exon expanding probes were purchased from Life Technologies (CA, USA), if not stated otherwise (Fbxo38 probe, Primerdesign Ltd, UK). The mean of threshold cycles (C_T_) of non-regulated mRNA expression of the housekeeping genes *gapdh* and *fbxo38* was used for normalization. The normalized mRNA expression of BLM-treated mice was standardized to the mean of the respective PBS-treated control group using the ΔΔC_T_ method.

### Lipid Extraction and Sphingolipid Analysis by LC-MS/MS

For the quantification of sphingolipids, skin tissue samples (approximately 5 mg each) were spiked with 150 µl water, 150 µl extraction buffer (citric acid 30 mM, disodium hydrogen phosphate 40 mM), 1,000 µl methanol/chloroform/hydrochloric acid (15:83:2, v/v/v), and 20 µl of the internal standard solution containing sphingosine-1-phosphate-d7 and C18:0-GluCer-d5 (both Avanti Polar Lipids, Alabaster, USA) and C24:0 Cer-d4 (Chiroblock GmbH, Bitterfeld-Wolfen, Germany) (400 ng/ml each). The mixture was homogenized using a swing mill (Mixer Mill MM 400, Retsch, Haan, Germany) and four zirconium oxide grinding balls per sample.

The lower organic phase was evaporated at 45°C under a gentle stream of nitrogen and reconstituted in 100 µl of tetrahydrofuran/water (9:1, v/v) with 0.2% formic acid and 10 mM ammonium formate. Afterward, amounts of sphingolipids were analyzed by liquid chromatography coupled to tandem mass spectrometry (LC-MS/MS). An Agilent 1100 series binary pump (Agilent technologies, Waldbronn, Germany) equipped with a Luna C8 column (150 mm × 2 mm ID, 3-µm particle size, 100 Å pore size; Phenomenex, Aschaffenburg, Germany) was used for chromatographic separation. The column temperature was 35°C. The high-performance liquid chromatography mobile phases consisted of water with 0.2% formic acid and 2 mM ammonium formate (mobile phase A) and acetonitrile/isopropanol/acetone (50:30:20, v/v/v) with 0.2% formic acid (mobile phase B). For separation, a gradient program was used at a flow rate of 0.3 ml/min. The initial buffer composition 55% (A)/45% (B) was held for 0.7 min and then within 4.0 min linearly changed to 0% (A)/100% (B) and held for 13.3 min. Subsequently, the composition was linearly changed within 1.0 min to 75% (A)/25% (B) and then held for another 2.0 min. The total running time was 21 min and the injection volume was 10 µl. To improve ionization, acetonitrile with 0.1% formic acid was infused post-column using an isocratic pump at a flow rate of 0.15 ml/min. After every sample, sample solvent was injected for washing the column with a 12-min run. At the end of the wash run, the initial chromatographic conditions of the analytical run were recovered. The MS/MS analysis was performed using a triple-quadrupole mass spectrometer API4000 (Sciex, Darmstadt, Germany) equipped with a Turbo V Ion Source operating in positive electrospray ionization mode. The MS parameters were set as follows: Ionspray voltage 5,500 V, ion source temperature 500°C, curtain gas 30 psi, collision gas 12 psi, nebulizer gas 40 psi, and heating gas 60 psi. The analysis was done in multiple reaction monitoring (MRM) mode.

Data acquisition was done using Analyst Software V 1.6 and quantification was performed with MultiQuant Software V 3.0 (both Sciex, Darmstadt, Germany), employing the internal standard method (isotope dilution mass spectrometry). Variations in accuracy of the calibration standards were less than 15% over the whole range of calibration, except for the lower limit of quantification, where a variation in accuracy of 20% was accepted.

### Statistical Analysis

Statistical analysis was carried out using SPSS 24 (Chicago, IL, USA). Depending on the number of comparative groups, data were analyzed with the *unpaired t-test* or *one-way ANOVAs with Bonferroni’s post hoc multi-comparison*. To assess changes toward the PBS control group used for normalization, we performed a *one sample t-test*. Results are presented as means ± SD using the software Graph Pad Prism 5 (La Jolla, CA, USA). Significant values are marked by hashtags (#/##/###) for comparisons to the respective controls or asterisks (*/**/***). Symbols represent *P*-values of *p* ≤ 0.05/*p* ≤ 0.01/*p* ≤ 0.001.

## Results

### Low-Dose BLM-Induced Skin Inflammation Results in Fibrotic Manifestations in WT and S1P5^−/−^ Mice

**Figure 2:**
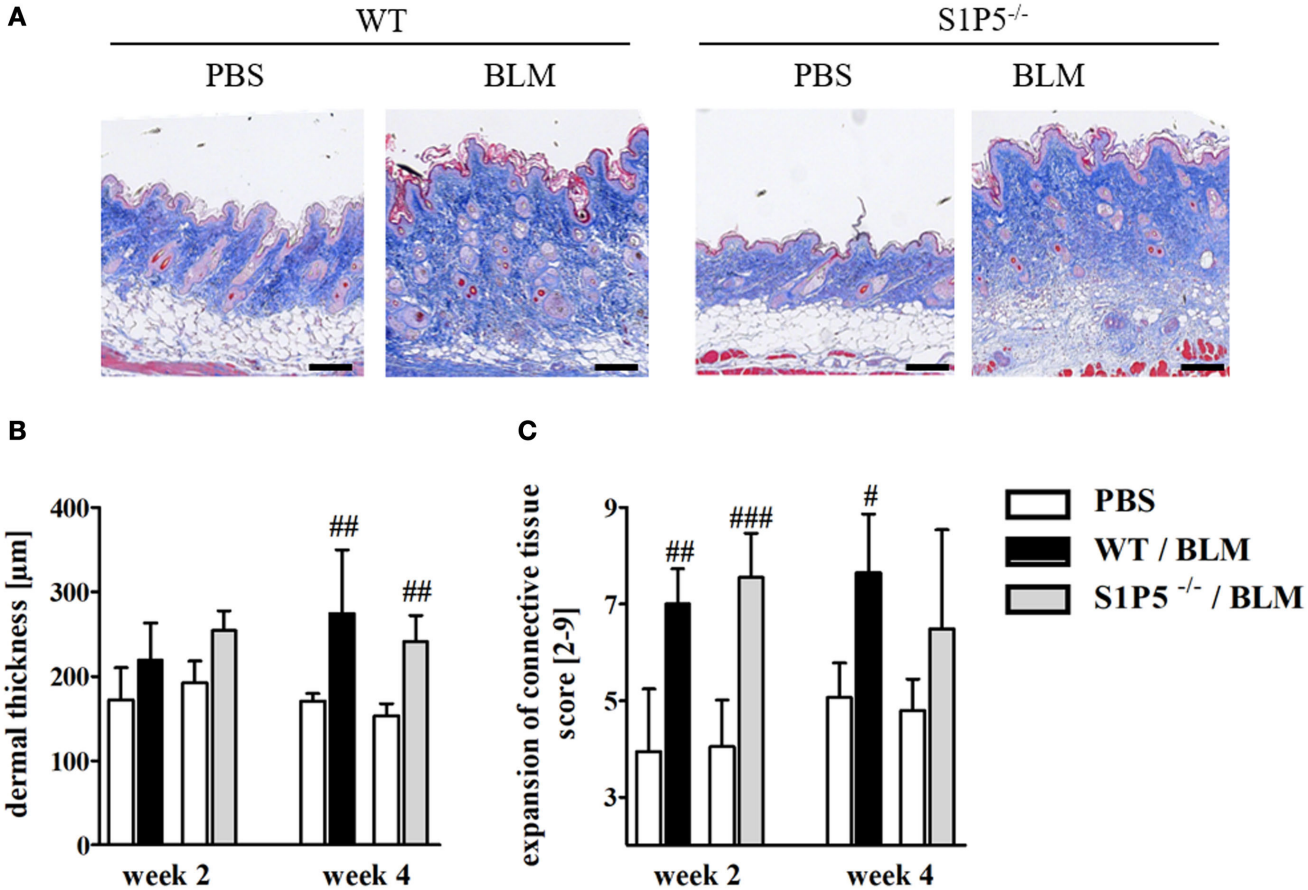
Low-dose BLM induces fibrotic alterations in the skin of WT and S1P5^−/−^ mice. **(A)** Representative skin sections of mice treated 4 weeks with BLM or PBS stained histochemically with Masson’s trichrome to indicate fibrotic alterations (scale bar = 100 µm, blue = connective tissue). **(B)** Dermal thickness and **(C)** expansion of connective tissue of WT and S1P5^−/−^ mice treated 2 and 4 weeks with BLM were determined in microscopic images of stained skin sections (see Materials and Methods). BLM-treated WT mice are represented as black bars, and S1P5^−/−^ mice are represented as gray bars. All results are shown as mean ± SD of *n* = 4–5 mice/group with # indicating *p* ≤ 0.05, ## for *p* ≤ 0.01 and ### for *p* ≤ 0.001, compared with the respective PBS controls (adjacent white bars). Statistical analysis was performed using a *one-way ANOVA with Bonferroni’s multiple comparison test*. ANOVA, analysis of variance; BLM, bleomycin; PBS, phosphate-buffered saline; WT, wild type.

**Figure 3:**
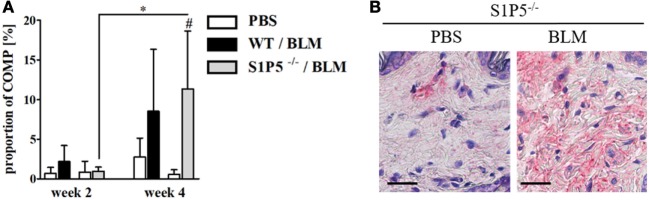
COMP expression increasing in the skin of S1P5^−/−^ mice after 4 weeks of low-dose BLM treatment. **(A)** Proportion of COMP within the dermis of WT and S1P5^−/−^ mice treated for 2 and 4 weeks with PBS or BLM was determined in microscopic images of COMP-stained skin sections (see Materials and Methods). BLM-treated WT mice are represented as black bars, and S1P5^−/−^ mice are treated as gray bars. **(B)** Representative microscopic pictures of skin sections from S1P5^−/−^ mice treated for 4 weeks with PBS or BLM. Immunohistochemically stained for COMP (scale bar = 20 µm, red = COMP). Results in **(A)** are shown as mean ± SD of *n* = 3–4 mice/group with #/* indicating *p* ≤ 0.05. Hashtags (#) characterize significance in comparison with the respective PBS controls (adjacent white bars). Statistical analysis was performed using a *one-way ANOVA with Bonferroni’s multiple comparison test*. ANOVA, analysis of variance; BLM, bleomycin; COMP, cartilage oligomeric matrix protein; PBS, phosphate-buffered saline; WT, wild type.

To generate an inflammatory milieu in the skin contributing to fibrosis, repetitive subcutaneous injections of low-dose BLM (25 μg/day; 5 days/week) either for a period of 2 or 4 weeks were applied in a mouse model of scleroderma. In both mouse groups, WT and S1P5^−/−^, the analysis of skin areas revealed a transcriptional upregulation of *ICAM-1, CCR2, CD206*, and *TGF-β1* in mice treated 2 weeks with BLM related to the respective PBS-injected control groups (Figures 1A–D). Simultaneously, a higher number of tissue-infiltrating cells was detected in 2-week BLM- versus PBS-treated skin areas (Figure 1E) whereby the quantity of α-SMA positive myofibroblasts within the dermis was not significantly altered (Figure S1 in Supplementary Material). Four weeks of BLM application caused a significant increase of dermal thickness, accompanied by an increased expansion of collagenous connective tissue in both genotypes (Figures 2A–C). Moreover, an augmented proportion of COMP within the dermis was found to be significant in the S1P5-deficient mice and in tendency in the WT mice following 4 weeks of BLM treatment (Figure 3). These data suggest a treatment-induced inflammatory and fibrogenic milieu in the skin of WT and S1P5^−/−^ mice, leading to comparable fibrotic alterations after 4 weeks.

### Low-Dose BLM Treatment Not Affecting the Quantity of CD31-Marked Blood Vessels in the Skin of WT and S1P5^−/−^ Mice

**Figure 4:**
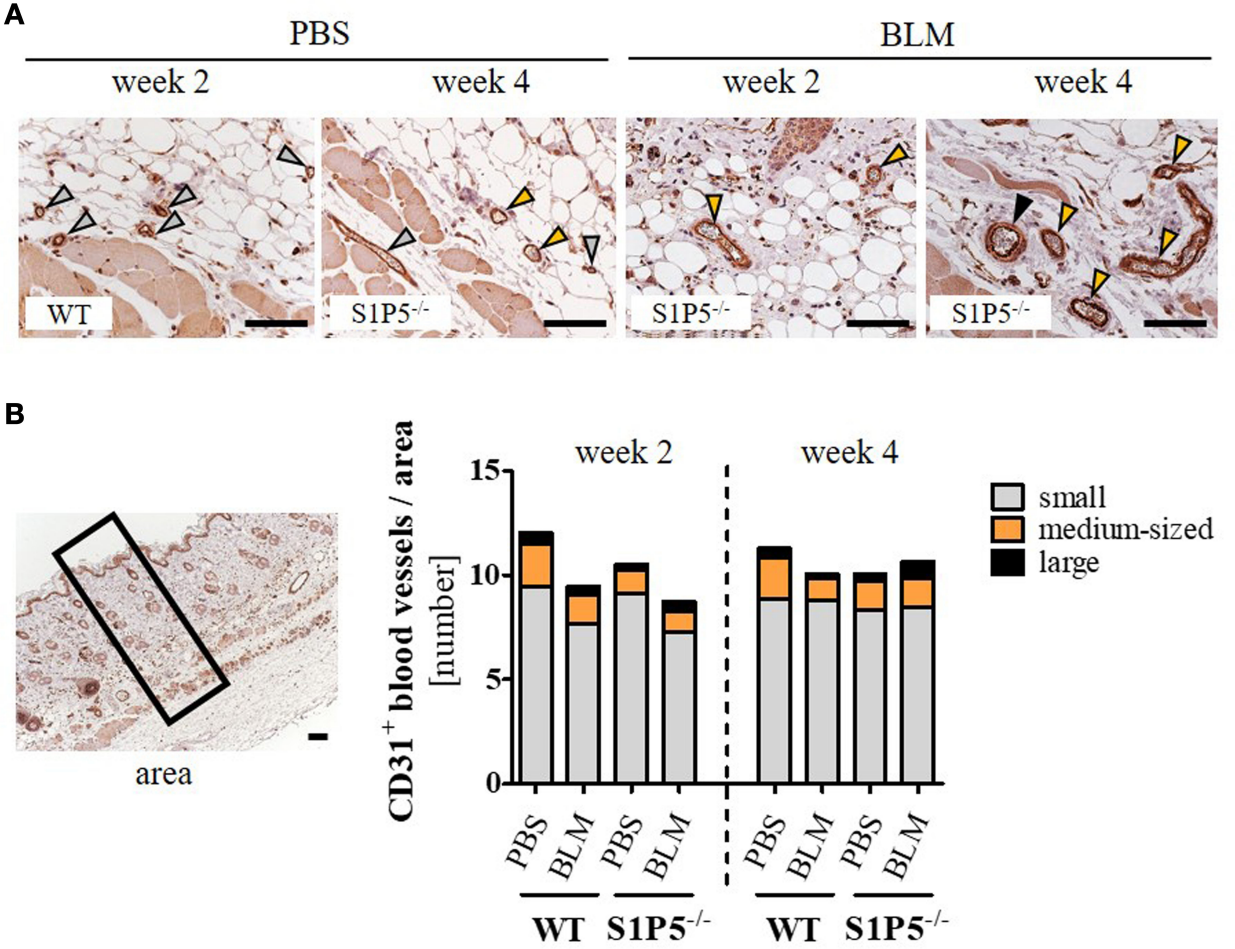
Quantity of CD31-positive blood vessels in the skin of WT and S1P5^−/−^ mice, which is not altered after low-dose BLM treatment. **(A)** Representative skin sections of mice stained for CD31 to mark blood vessels of different sizes, indicated by specifically colored arrows (gray arrow = small vessel; orange arrow = medium-sized vessel; black arrow = large vessel; scale bar = 50 µm). **(B)** Small, medium-sized, and large CD31-positive blood vessels in comparable skin areas of 2- and 4-week-treated WT and S1P5^−/−^ mice were counted manually on a microscope [visual definition of blood vessel sizes: see **(A)**; representation of an area: see left]. Small vessels are represented as gray parts, medium-sized vessels as orange parts, and large vessels as black parts of the stacked bars. Result is shown as mean of 3–6 areas/mice of *n* = 4–5 mice/group. Statistical analysis was performed using a *one-way ANOVA with Bonferroni’s multiple comparison test*. ANOVA, analysis of variance; BLM, bleomycin; WT, wild type.

Vasculopathy, especially the loss of small capillaries, is a characteristic of SSc. Therefore, we immunohistochemically assessed the effect of low-dose BLM application on the number of differentially sized blood vessels in the skin of WT- and S1P5-deficient mice. As a result, blood vessels stained by the endothelial marker CD31 did not differ significantly neither between the genotypes, nor between PBS and BLM treatment or between the points in time (Figure 4).

### S1P5 Deficiency Augmenting a Different Long-Chain Ceramide Profile in the Skin after BLM Treatment

**Figure 5:**
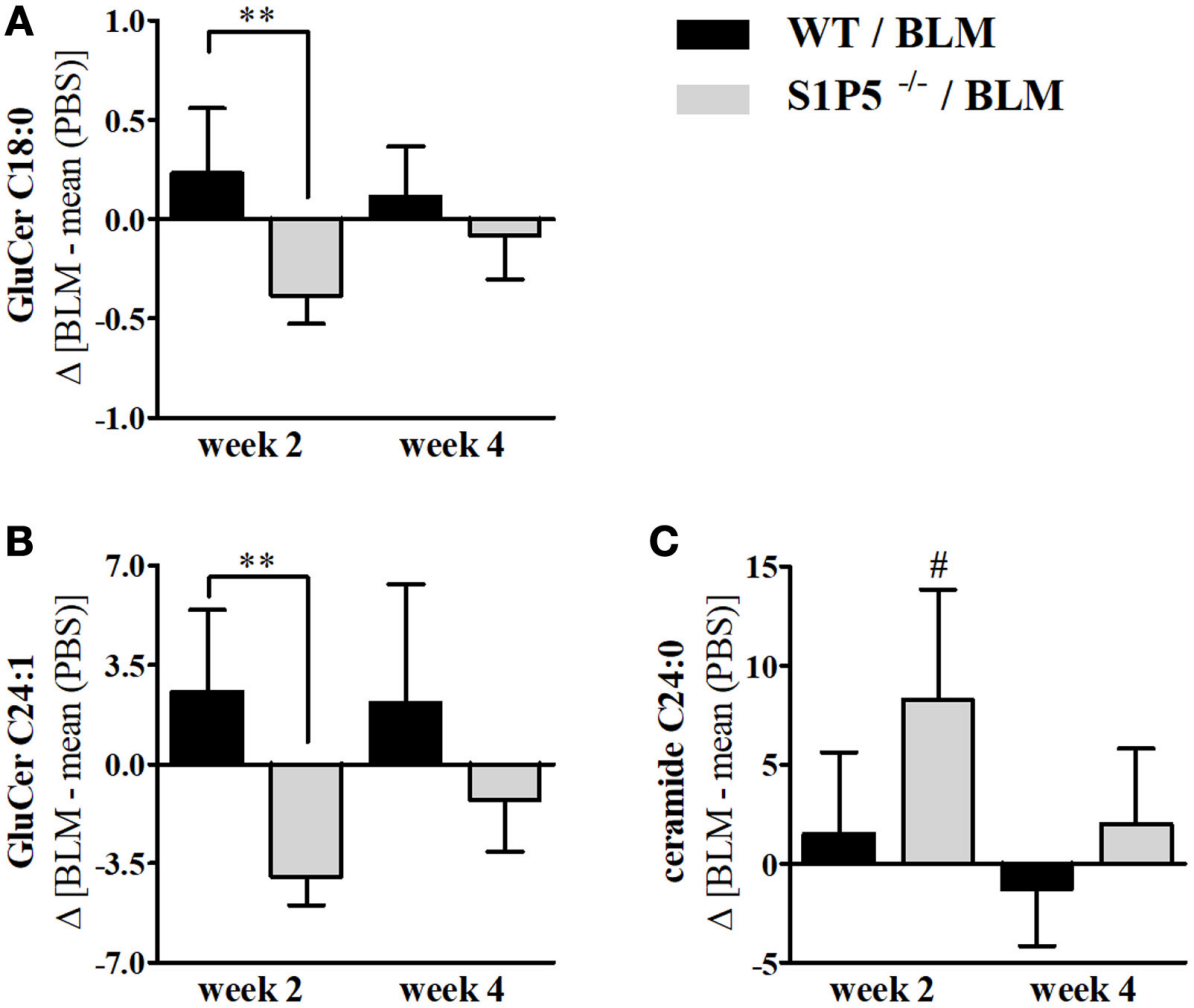
Long-chain ceramides, showing a decrease in S1P5^−/−^ following BLM treatment. BLM-induced regulation of GluCer C18:0 **(A)**, GluCer C24:1 **(B)**, and ceramide C24:0 **(C)** in the skin of WT (black bars) and S1P5^−/−^ mice (gray bars). Lipid measurements were performed after extraction by LC-MS/MS analysis (ng/mg) and presented here for BLM-treated mouse groups as difference to the mean of respective PBS controls. Data are shown as mean ± SD of *n* = 4–5 mice/group with # indicating *p* ≤ 0.05, ** for *p* ≤ 0.01. Hashtags (#) characterize significance in comparison to the respective PBS controls. Statistical analysis was performed using a *one-way ANOVA with Bonferroni’s multiple comparison test*. (Δ = difference). ANOVA, analysis of variance; BLM, bleomycin; GluCer, glucosylceramide; LC-MS/MS, liquid chromatography coupled to tandem mass spectrometry; PBS, phosphate-buffered saline; WT, wild type.

Effects of low-dose BLM-induced alterations on S1P and dhS1P concentrations, as well as on other lipids relevant for skin barrier function, were determined by mass-spectrometric lipid analysis of skin tissue samples. Dermal S1P and dhS1P levels were detectable albeit below the lower limit of reliable quantification. Corresponding analysis of the mRNA expression of S1P metabolizing enzymes, including S1P lyase, S1P phosphatase isoforms, as well as sphingosine kinase isoforms, in skin tissues revealed no significant differences between the genotypes (Figure S2 in Supplementary Material). Interestingly, determination of the local levels of characteristic ceramide species of the skin in BLM- versus PBS-treated mice demonstrated elevated concentrations of GluCer C18:0 and C24:1 in the WT mice. In contrast, significantly lower levels were measured in S1P5-deficient mice (Figures 5A,B). The significant reduction of ceramide compounds in S1P5-deficient mice compared with WT mice, together with the rise of ceramide C24:0 in knockout mice (Figure 5C), implies the involvement of S1P5 on local lipid composition in early BLM-induced skin inflammation.

### S1P5 Deficiency Affecting the Type of BLM-Induced Skin Inflammation by Preventing an Increase of GATA-3 and S1P3 mRNA Expression

**Figure 6:**
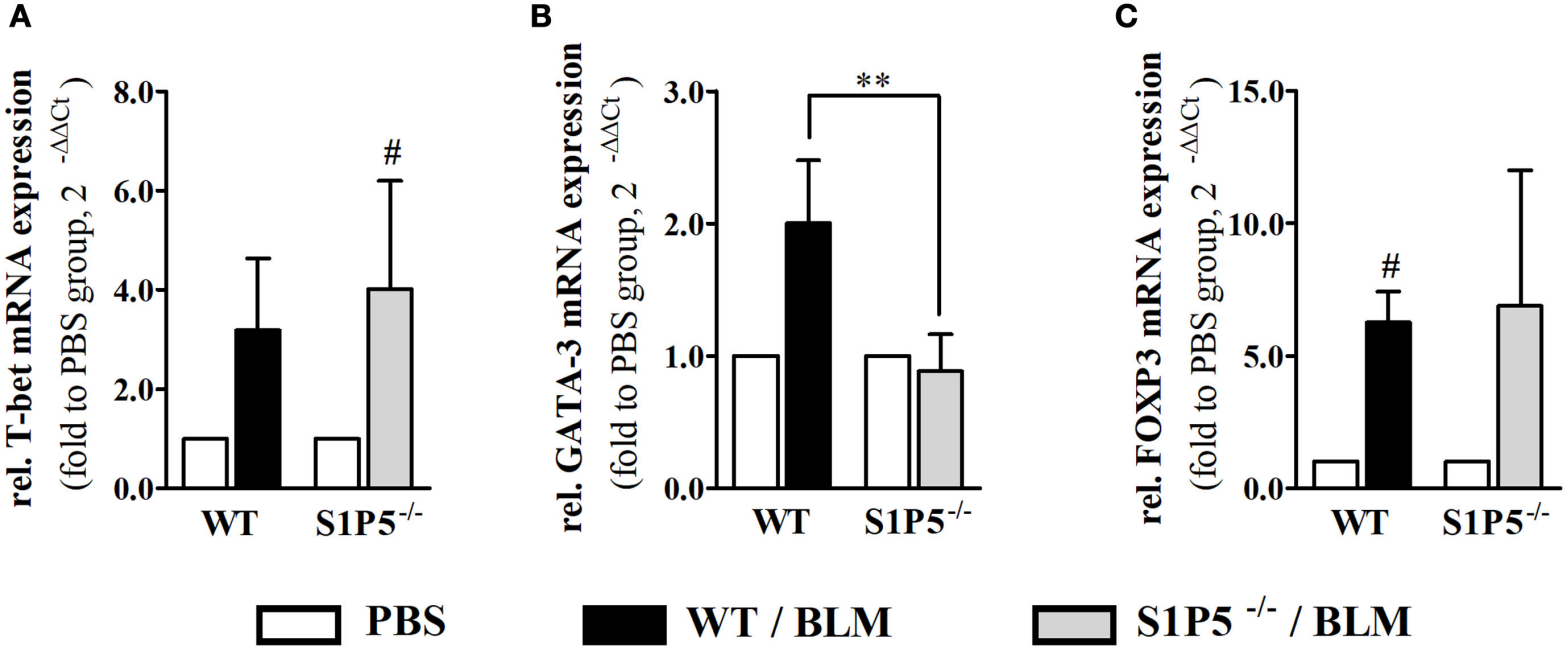
S1P5-dependent increase in GATA-3 transcripts after a 2-week low-dose BLM treatment. Relative mRNA expression of **(A)** *T-bet*, **(B)** *GATA-3*, and **(C)** *FOXP3* in skin tissue of BLM-treated WT (black bars) and S1P5^−/−^ (gray bars) mice. mRNA expression levels were determined by qRT-PCR analysis. All data are presented as fold change to the mean of the respective PBS controls and shown as mean ± SD of *n* = 3–5 mice/group with # indicating *p* ≤ 0.05 and ** for *p* ≤ 0.01. Hashtags (#) characterize significance in comparison to the respective PBS controls (adjacent white bars). Statistical analysis was performed using a *one sample t-test* for # or an *unpaired t-test* for *. (rel. = relative). BLM, bleomycin; PBS, phosphate-buffered saline; WT, wild type.

**Figure 7:**
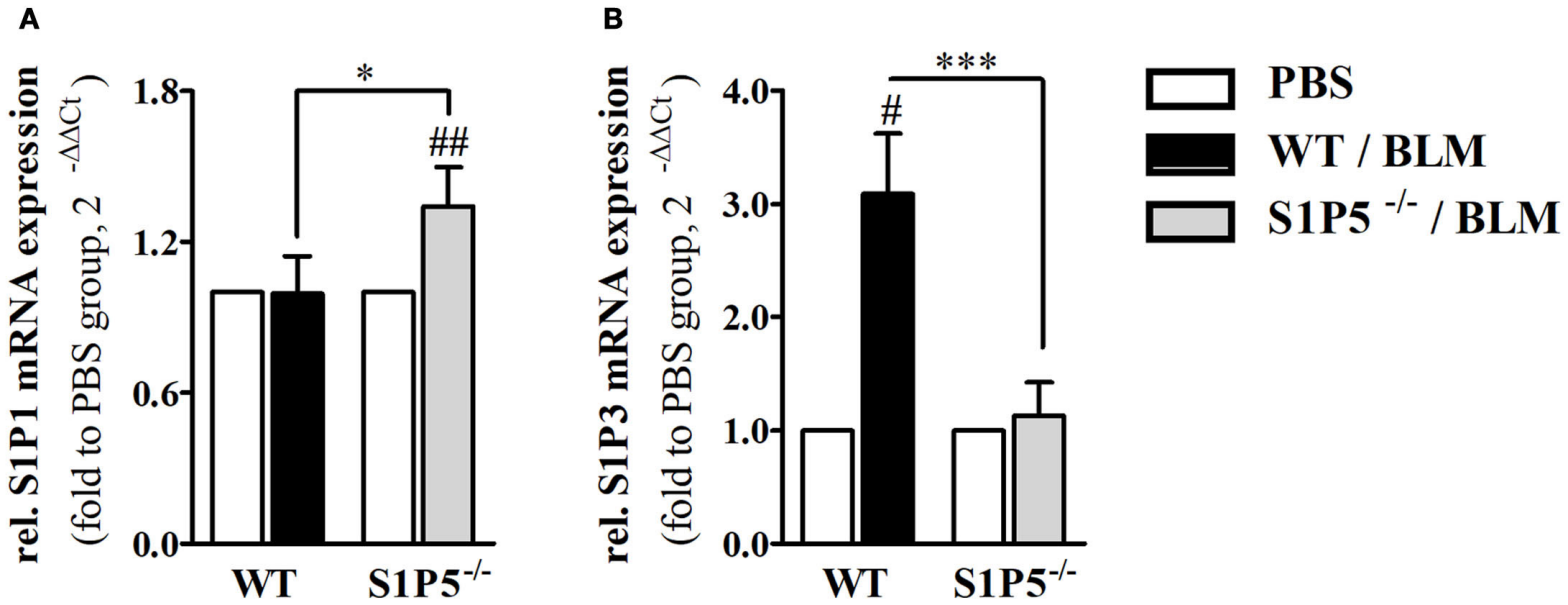
S1P5-dependent regulation of *S1P1* and *S1P3* mRNA in BLM-treated skin. Relative mRNA expression of **(A)** *S1P1* and **(B)** *S1P3* in skin tissue of BLM-treated WT (black bars) and S1P5^−/−^ (gray bars) mice. mRNA expression levels were determined by qRT-PCR analysis. All data are presented as fold change to the mean of the respective PBS controls and shown as mean ± SD of *n* = 3–5 mice/group with #/* indicating *p* ≤ 0.05, ## for *p* ≤ 0.01 and *** for *p* ≤ 0.001. Hashtags (#) characterize significance in comparison to the respective PBS controls (adjacent white bars). Statistical analysis was performed using a *one sample t-test* for # or an *unpaired t-test* for *. (rel. = relative). BLM, bleomycin; PBS, phosphate-buffered saline; qRT-PCR, quantitative real-time polymerase chain reaction; WT, wild type.

Migration of immune cells and T cell polarization play an important role for immune dysfunction observed in SSc. Therefore, S1P5-dependent, BLM-induced variations in the mRNA induction of relevant factors, which are expressed by immune cells and might be important for the pathogenesis of scleroderma, were examined. As a result, we found that the transcriptional expression of *T-bet* and *FoxP3* was equally induced in both genotypes (Figures 6A,C). Conversely, the BLM-induced regulation of Th2 characteristic transcription factor *GATA-3* mRNA differed significantly and was enhanced in the WT versus S1P5^−/−^ mice (WT: 2.0 ± 0.5-fold; S1P5^−/−^: 0.9 ± 0.3-fold) (Figure 6B). In parallel to the upregulation of *GATA-3* in treated WT mice, *S1P3* mRNA expression was significantly increased in WT compared with S1P5-deficient mice (Figure 7B). Different from *S1P3*, upregulated *S1P1* mRNA was detected in the skin of BLM-treated S1P5^−/−^ mice, however not in the WT mice (Figure 7A).

### S1P5 Deficiency Affecting Parameters of Skin-Fibrotic Processes

**Figure 8:**
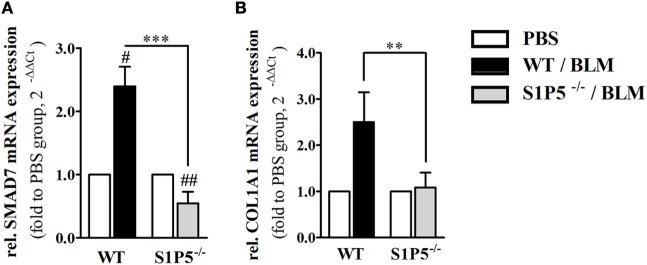
S1P5 impacts on SMAD7 and COL1A1 mRNA expression. Relative mRNA expression of **(A)** *SMAD7* and **(B)** *Col1A1* in skin tissue of BLM-treated WT (black bars) and S1P5^−/−^ (gray bars) mice. mRNA expression levels were determined by qRT-PCR analysis. All data are presented as fold change to the mean of the respective PBS controls and shown as mean ± SD of *n* = 3–5 mice/group with # indicating *p* ≤ 0.05, ##/** for *p* ≤ 0.01 and *** for *p* ≤ 0.001. Hashtags (#) characterize significance in comparison to the respective PBS controls (adjacent white bars). Statistical analysis was performed using a *one sample t-test* for # or an *unpaired t-test* for *. (rel. = relative). BLM, bleomycin; PBS, phosphate-buffered saline; qRT-PCR, quantitative real-time polymerase chain reaction; WT, wild type.

In order to elucidate whether S1P5 plays a role for TGF-β1 signaling, we determined mRNA expression of the pro-fibrotic TGF-β responsive gene *COL1A1* (42) as well as transcription of the inhibitory subunit in the TGF-β signaling pathway, SMAD7. After 2 weeks of subcutaneous BLM application, a trend to higher *TGF-β1* mRNA expression was measured in both genotypes (WT: 1.7 ± 0.2-fold; S1P5^−/−^: 1.9 ± 0.4-fold; Figure 1D). Differently, the TGF-β downstream *COL1A1* mRNA was exclusively upregulated in WT following 2 weeks of BLM treatment (WT: 2.5 ± 0.7-fold; S1P5^−/−^: 1.1 ± 0.3-fold; Figure 8B). Interestingly, *SMAD7* mRNA increased in WT, but was slightly diminished in S1P5-deficient mice after BLM application (Figure 8A).

## Discussion

To date, S1P5 function in general is still poorly characterized. It is well known that S1P5 affects the immune quiescence of the brain endothelial barrier, as well as the trafficking of natural killer cells (23, 40, 43). Furthermore, enhanced S1P5 activity was shown to influence proliferation, migration, and autophagy in human cancer cells (22, 44). Nevertheless, data concerning fibrotic S1P5 involvement are rare and ambiguous. Wünsche et al. imply a pro-fibrotic function of S1P5 in human mesangial cells (25), whereas others found that S1P5 mediates anti-fibrotic activities of the structural S1P-analogon FTY720 under skin-fibrotic conditions (45). Although the receptor S1P5 is expressed in the skin (24, 45), its dermatological function during inflammation and fibrogenesis has not been elucidated so far.

Microangiopathy, cutaneous inflammation, and fibrotic processes are important elements triggering the pathogenesis of SSc (2). In order to investigate S1P5 function in early-stage processes driving fibrogenesis, low-dose BLM injections were used in an SSc mouse model to induce fibrotic processes in the skin of WT and S1P5^−/−^ mice. By 2 weeks of subcutaneous BLM treatment (25 μg/day, 5 days/week), we generated an inflammatory and fibrogenic milieu in the skin of both genotypes. This became evident by a transcriptional increase of the corresponding markers *ICAM-1* (46–48), *CCR2* (49–52), *CD206* as marker for M2 macrophages (53, 54), and *TGF-β1* (55), along with significant fibrotic alterations at 4-week BLM-injected mice (Figures 1 and 2). Under our experimental conditions, skin thickening was less pronounced than in other SSc studies using 100 µg BLM and more in a 4-week treatment protocol (56, 57). Furthermore, a BLM-induced accumulation of α-SMA expressing myofibroblasts (58, 59) was not detectable upon our low-dose BLM treatment (Figure S1 in Supplementary Material). Nevertheless, the above-mentioned key factors for the initiation of fibrogenesis that are associated with the BLM model and/or the disease were transcriptionally induced after 2 weeks of low-dose BLM treatment. Enhanced ICAM-1 mediates leukocyte migration into inflamed areas (60–62) and has even shown to be upregulated in response to higher dose BLM injections (63, 64). Effects of 2-week low-dose BLM-induced mRNA expression of inflammatory factors were correlating to an increased number of infiltrated cells into BLM-treated tissues compared with the PBS controls (Figure 1E). Therefore, we have succeeded in our aim to mimic a moderate inflammatory and pro-fibrotic milieu of early-stage fibrogenesis within the skin of 2-week BLM-treated WT and S1P5-deficient mice.

Sphingolipids and ceramides are fundamental for constitution of the cell membrane and for epithelial barrier function. Even more, some ceramide species are regulators of key physiological functions such as migration, proliferation, and apoptosis (65, 66), and thus may bias inflammation. In 2-week BLM-treated skin areas, S1P5 augmented a different long-chain ceramide profile with an increase of GluCer C18:0 and GluCer C24:1 in the WT, and a decrease in the S1P5-deficient mice (Figures 5A,B). Interestingly, already a small accumulation of GluCers has been shown to trigger inflammation in glucocerebrosidase-deficient mice (39). In parallel, ceramide C24:0 was especially induced in the above-mentioned S1P5^−/−^ group (Figure 5C), whereas the shorter chain ceramide C16:0 was not significantly altered following BLM treatment (data not shown). Within the Langmuir model, Pullmanova et al. recently demonstrated that the proportion of Cer C24 compared with shorter Cer C16 is important to maintain barrier permeability in the outmost layer of human epidermis (67). Therefore, the BLM-induced increase in Cer C24:0 in S1P5-deficient mice might improve barrier function. Whatsoever are the mechanisms underlying the observed effects, these results suggest a potentially supportive role of S1P5 for triggering inflammatory processes in the applied SSc mouse model.

A Th2-biased immune response induces tissue fibrosis and is implicated in SSc pathogenesis (33). According to our results, S1P5 participates significantly in the transcriptional induction of Th2-characteristic transcription factor *GATA-3* under BLM-induced inflammatory conditions (Figures 1 and 4). The pathophysiological relevance of GATA-3 has been proven by several studies. Referred to those, the upregulation of GATA-3 in T cells increases IL-13 synthesis (68, 69). Especially IL-13, produced by CD8+CD28− scleroderma T cells, enhances COL1A1 protein expression in dermal fibroblasts (70). We, respectively, detected an induction of *COL1A1* transcripts, which was, similar to *GATA-3*, exclusively found in inflammatory skin tissues of S1P5-expressing WT mice (Figure 7). Besides *GATA-3*, intriguingly, *S1P1* and *S1P3* mRNA was differentially upregulated in the genotypes after 2 weeks of BLM treatment (Figure 7). We hypothesize that transcripts were introduced by infiltrating cells into inflamed skin tissues and therefore point to a different immunological composition of the inflammatory infiltrates in WT and S1P5-deficient mice. This suggests a modulating effect of cutaneous S1P5 expression on the quality of inflammation. Interestingly, S1P3 was shown to support inflammation as well as fibrosis (71). In previous studies, we were able to show that S1P3 mediates pro-fibrotic differentiation of myofibroblasts through a TGF-β/SMAD-dependent pathway (72) and also others stated a fibrogenic involvement of S1P3 (73).

To get an impression of S1P5 implications concerning fibrotic TGF-β/SMAD signaling in the 2-week-treated skin tissues, we analyzed the mRNA expression of selected TGF-β downstream targets as well as the regulatory subunit SMAD7 (29, 74, 75). Impairment of SMAD7 signaling has been associated with scleroderma fibroblasts (76). *TGF-β1*expression of PBS-treated controls does not differ between the genotypes (data not shown) and BLM-induced *TGF-β1* upregulation was comparable in WT and S1P5-deficient mice (Figure 1). Although there were no significant differences referring to the myofibroblast marker *α-SMA* among WT and S1P5^−/−^ mice (mRNA data not shown), we observed a specific transcriptional increase of *COL1A1* upon BLM treatment. Similar to the fibrogenic upregulation of *GATA-3* and *S1P3* transcripts, this effect was limited to the 2-week BLM-treated WT mice. Remarkably, a simultaneous increase of regulatory *SMAD7* was observed in this experimental group (Figure 8). Since SMAD7 protein is known to control intracellular TGFβ-induced SMAD signaling, regulating its activity is very important. Therefore, *SMAD7* mRNA induction in the WT group might be a feedback of low-protein level. In contrast, *SMAD7* transcription was repressed in the skin of 2-week BLM-treated S1P5-deficient mice, possibly reflecting high-protein level. In summary, our data emphasize a supportive S1P5 effect on early-stage fibrotic processes.

Subsequently, fibrotic quality within the dermal extracellular matrix was evaluated with respect to the TGF-β-inducible collagen-crosslinking protein COMP (31, 77). COMP served as a fibrotic marker (78) and its overexpression has been shown in SSc (31, 79). Immunohistological COMP analysis revealed an increase in the proportion of COMP within the dermis of 4-week BLM-treated skin areas compared with those injected with PBS. This effect was detectable as a trend in the WT and significantly in the S1P5-deficient mice (Figure 3). Therefore, enhanced COMP expression supports the finding of moderate fibrotic skin thickening at 4-week BLM-treated S1P5^−/−^ mice. The extent in COMP induction varies between WT and S1P5-deficient mice. This suggests that the experimental groups were in a different stage during fibrogenesis. Therefore, S1P5 expression may impact on disease progression.

Disturbed angiogenesis and impaired vasculogenesis are primary events leading to the microangiopathy seen in SSc (80–82). Since it is unclear whether BLM treatment reduces the number of capillaries (83), we wanted to get an impression if the applied BLM model reflected the loss of small blood vessels in the injected skin areas of WT and S1P5-deficient mice. Our results reveal no significant differences regarding the number of CD31-stained blood vessels (Figure 4). In accordance with other publications, repetitive subcutaneous BLM application seems not to mimic the complex vascular damage described in SSc vasculopathy (84).

In order to identify specific therapeutic options, it is important to gain a better understanding of early molecular and cellular cutaneous processes and regulators, predicting development and progress of SSc pathogenesis. To date nothing is known about S1P5 participation concerning this matter. Our data, generated in a modified mouse model of the inflammatory fibrotic disorder, proposed that S1P5 deficiency impacts on early-stage dermal inflammatory responses by affecting long-chain ceramide profiles in the skin as well as the composition of cellular infiltrates. A transient pro-fibrotic S1P5 contribution is indicated by our thorough pilot study; however, more extended studies have to be considered. In conclusion, for the first time the present study linked S1P receptor 5 to early stages within the development of inflammatory fibrotic skin alterations that may encourage the ongoing search for new therapeutic options for SSc patients.

## Ethics Statement

The animals were bred and fed under pathogen-free conditions in secluded scantainers. All animal experiments were performed in accordance with the German animal welfare law and had been declared to the Animal Welfare Officer as the chairperson of the ethical oversight committee of the Goethe University Frankfurt/Main. The animal housing facility was licensed by the local authorities of the Regierungspraesidium Darmstadt (Protocol no. Az: 32.62.1). The methods used to euthanize the animals humanely were consistent with the recommendations of the AVMA Guidelines for the Euthanasia of Animals.

## Author Contributions

KS analyzed all data, wrote the manuscript, performed statistics, and designed the figures. ST and DT performed the LC-MS/MS measurement of sphingolipids. LB established and performed the COMP staining. MJ, AS, and FO helped by conduction of the animal experiment and with connective tissue scoring. FZ supported the COMP evaluation and helped with data interpretation. JP supplied basic lab equipment. HR had the idea, acquired the funding, designed and supervised all experiments, checked the data, and finalized the manuscript.

## Conflict of Interest Statement

The authors declare that the research was conducted in the absence of any commercial or financial relationships that could be construed as a potential conflict of interest.

## Abbreviations

dhS1P, dihydrosphingosine-1-phosphate; FoxP3, forkhead box P3; GATA-3, GATA binding protein 3; Tbet, Tbx21; Th, T helper cell.
