# Supplementary material for: Sphingosine-1-Phosphate Receptor 5 Modulates Early-Stage Processes during Fibrogenesis in a Mouse Model of Systemic Sclerosis: A Pilot Study
Source: Front Immunol. 2017 Sep 29;8:1242. doi: 10.3389/fimmu.2017.01242 (PMC5626866; doi:10.3389/fimmu.2017.01242)
Supplement: Supplementary file 1 [file Data_Sheet_1.DOCX]

Supplementary Material

Sphingosine-1-Phosphate Receptor 5 Modulates Early-Stage Processes during Fibrogenesis in a Mouse Model of Systemic Sclerosis: a Pilot Study

Katrin G. Schmidt, Martina Herrero San Juan, Sandra Trautmann, Lucija Berninger, Anja Schwiebs, Florian Ottenlinger, Dominique Thomas, Frank Zaucke, Josef M. Pfeilschifter and Heinfried H. Radeke^*^

*** Correspondence:** Prof. Dr. med. Heinfried H. Radeke: [radeke@em.uni-frankfurt.de](mailto:radeke@em.uni-frankfurt.de)

##
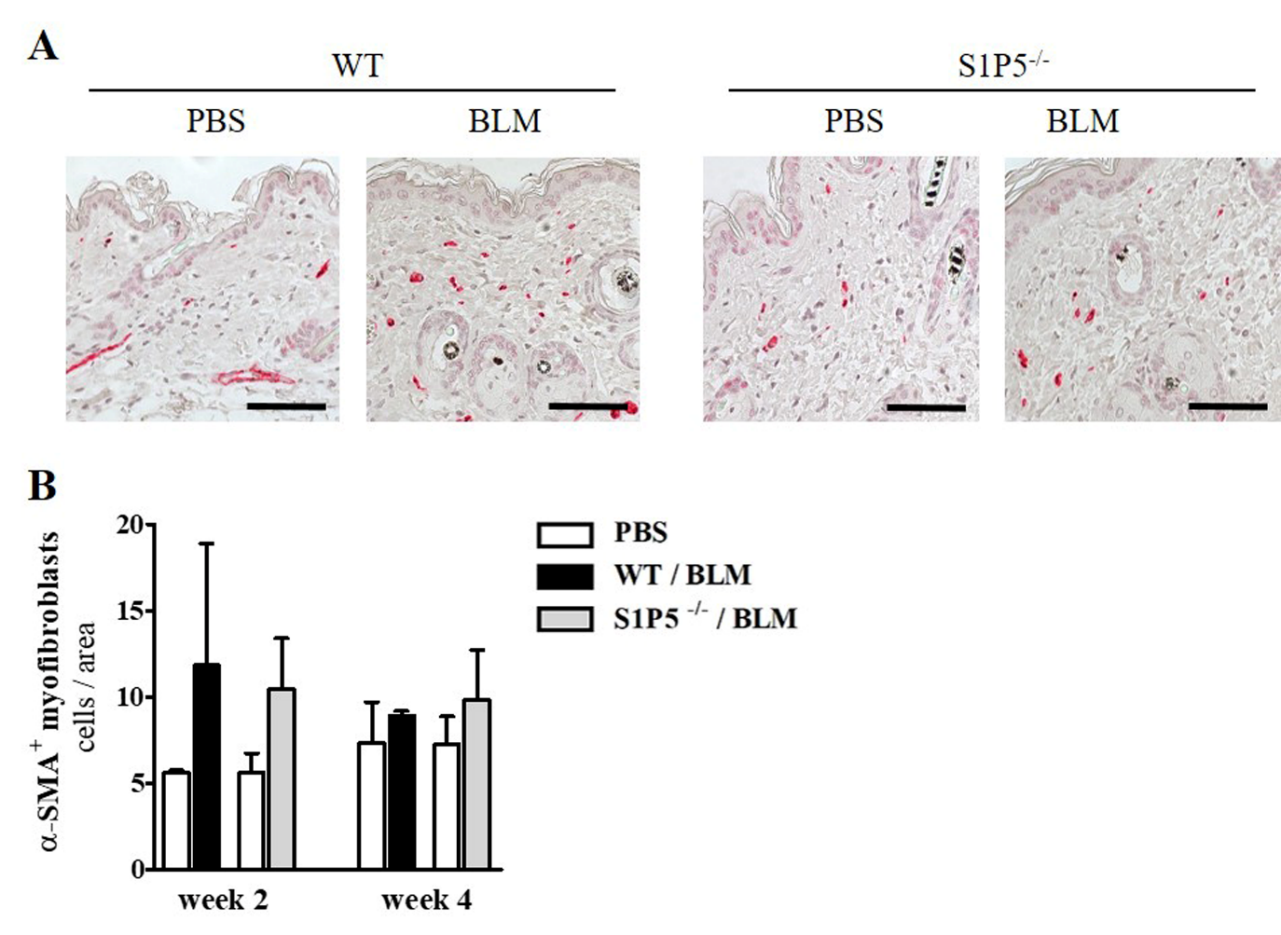


**Supplementary Figure 1.** Number of α-SMA-positive myofibroblasts within the dermis of WT and S1P5^-/-^ mice is not altered after low-dose BLM treatment. **(A)** Representative image areas of skin sections from WT and S1P5^-/-^ mice treated for 2 weeks with PBS or BLM. Immunohistochemically stained for α-SMA (scale bar = 50 μm, red = α-SMA). **(B)** α-SMA-positive myofibroblasts within the dermis of WT and S1P5^-/-^ mice treated for 2 and 4 weeks with PBS (adjacent white bars) or BLM were counted manually on a microscope (200x magnification). BLM-treated WT mice are represented as black, S1P5^-/-^ mice as gray bars. Results in B are shown as mean of 5-8 areas/mice of n = 3-5 mice/group. Statistical analysis was performed using a *one-way ANOVA with Bonferroni´s multiple comparison test*.


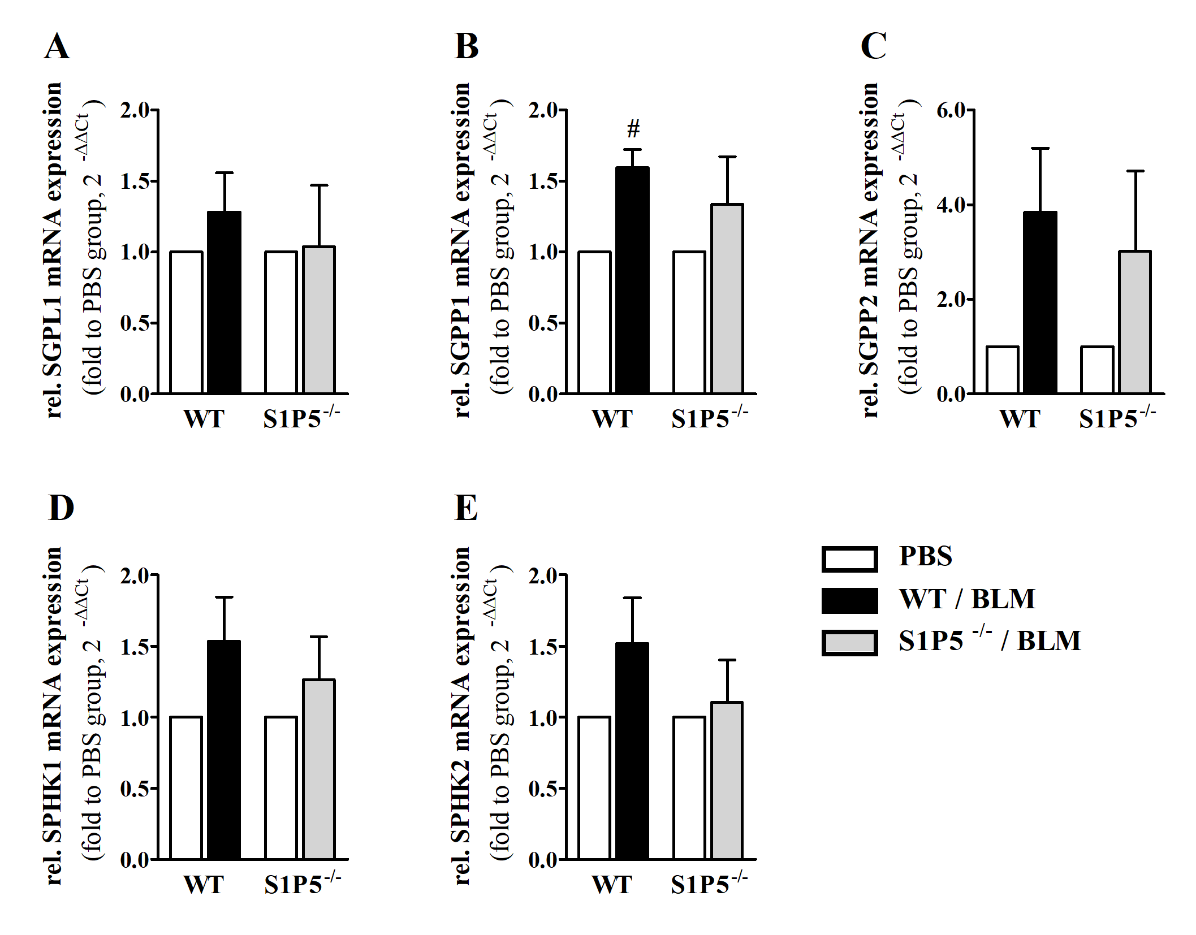


**Supplementary Figure 1.** Gene expression of S1P metabolizing enzymes in the skin of treated WT and S1P5^-/-^ mice. Relative mRNA-expression of **(A) s**phingosine-1-phosphate lyase (*SGPL1*), **(B, C)** Sphingosine-1-phosphate phosphatase isoforms 1 & 2 (*SGPP1, SGPP2*) **(D, E)** Sphingosine kinase isoforms 1 & 2 (*SPHK1, SPHK2*) in the skin of BLM-treated WT (black bars) and S1P5^-/-^ (gray bars) mice. mRNA expression levels were determined by qRT-PCR analysis. All data are presented as fold change compared to the mean of the respective PBS controls and shown as mean ± SD of n = 3-5 mice/group with # indicating p ≤ 0.05, compared to the respective PBS controls (adjacent white bars). Statistical analysis was performed using a *one sample t-test.* (rel. = relative, BLM = bleomycin)
